# Supplementary material for: Learning curve and surgical outcome of robotic assisted colorectal surgery with ERAS program
Source: Sci Rep. 2022 Nov 29;12:20566. doi: 10.1038/s41598-022-24665-w (PMC9709162; doi:10.1038/s41598-022-24665-w)
Supplement: Supplementary file 2 — Supplementary Information 2. [file 41598_2022_24665_MOESM2_ESM.zip › RAL Raw data-Ñ[▒K/136/HS_ñw╝╨░O▒Kñσ.pdf]

正式摘要(電子簽章：000000234324839)

W102 / W102 ☐英文標題(注意:無英文姓名-請補病人基本檔!) ☐【讀取】憑證

臺中榮民總醫院
出院病歷摘要

轉入號： 轉出號：

[redacted]

(8)流水編號	
(9)入院日期	110/12/26 (2021)
(10)轉科日期	
(11)出院日期	111/01/05 (2022) 住院天數計 10 日

診斷

- 入院：1. rectum cancer, adenocarcinoma, cT3N0M0, stage IIA, s/p neoadjuvant CCRT
- 出院：rectum cancer, 4 cm FAV anterior wall, adenocarcinoma, cT3N2M0, stage IIIB,
  s/p SCRT + FOLFOX*6 2021/8-2021/10
  with partial response cT3N0M0
  s/p robotic LAR TME, Lt plevic lymphadenectomy + ileostomy on 2021/12/29
  ypT0N0(0/18), CRM-, DisMg: -1.5cm, TRG0

主訴

- constipation for months and body weight loss(4Kg)

病史

- This 40 years old male patient has a past history of rectum cancer, adenocarcinoma, cT3N0M0, stage IIA, s/p neoadjuvant CCRT.
  He had a constipation for months and body weight loss(4Kg). Then, he went to 802 hospital. Rectum cancer was diagnosised there. Neoadjuvant CCRTY was done there. They suggested operation. However, he hesitated due to risk. The doctor there suggested further visited to our hospital for operation. Under the impresssion of rectum cancer, he was admitted for op.

體檢發現

-
  - General appearance :
    - easy looking , conscious clear , without cardiopulmonary distress
    - Vital signs自動帶入date 體溫(BT) 脈搏(P) 呼吸(R) 血壓(BP) 身高(BH) 體重(BW) BMI
      20211226 35.5℃ 89次/分 18次/分 115/78mmHg 172cm 57.7kg 19.5
  - HEENT :
    - Eyes: no anemic conjunctiva, no icteric sclera, normal EOM
    - ENT: no otorrhea, no rhinorrhea, no erythematous mucosa, no injected tonsil, no deformity
  - Neck: supple, freely movable, trachea midline,no deformity, no engorged jugular vein , no palpable thyroid or lymph node enlargement
  - Chest:
    - Inspection: Grossly normal, symmetric and fully expansion, no accessory muscle used, no operation scar, no spider angiomata, no gynecomastia
    - Palpation: no palpable mass or nodule, no subcutaneous emphysema, no tenderness
    - Ausculation: clear breathing sound
  - Heart:
    - Percussion: normal heart size
    - Ausculation: regular heart beat, normal S1, S2, no gallop, no murmur
  - Abdomen:
    - Inspection: in
      - flat contour, symetrically, no scar, no stria, no dilated veins, no rashes
    - Ausculation: normoactive bowel sound, no abdominal bruits
    - palpation: soft, no muscle guarding or rigidity, no local tenderness, no rebounding pain, no palpable mass, no palpable pulsation
    - Percussion: no shifting dulllness, no hepatomegaly, no splenomagaly
  -

Peripheral pulsation :	CA	BA	RA	FA	PA	PTA	DPA
Right side :	++	++	++	++	++	++	++/+++
Left side :	++	++	++	++	++	++	++/+++

  - Genito-anus: no tenderness or palpable mass lesion or rectal shelf, normal external genitalia, normal anal tone, brown stool

CRS CONDITION

- CEA: 1.66 ng/ml
-
  - Lung

手術日期及方法

- 手術日期及方法20211229 Lymphadenectomy, pelvic Ileostomy
  20211229 1. Robotic (XI) assisted low anterior resection TME
  2. Lt side pelvic LN dissection
  3. protective ileostomy.

住院治療經過

- After admission, we arrange pre-op survey. Sigmoidoscopy and plevis MRI was done. Lab data showed no contraindication for op. Robotic LAR was performed on 2021/12/29. After op, he can tolerated soft diet now. Wound pain was tolerated. Under stable condtiion, he was discharged and arranged OPD f/u.

合併症

一般檢查

- 累積報告 :

DATE	TIME	ALB	TP	BIL.T	BIL.D	ALKP	AST	ALT	LDH	AMY	LIPASE	NH3	Ferritin	UA
1101225	1559	4.6						22						
1101227	1245			0.5	0.1									
1101230	1122	3.3					14	9						
1110103	0915	3.6		0.3	0.1		19	8						

DATE	TIME	WBC	RBC	HGB	HCT	MCV	PLT	BLST	NEUT	BAND	SEG	LYM	MON	EOS	BAS
1101225	1559	6430		14.9			221		79.4			12.6	5.6	1.9	0.5
1101230	1108	8930	3.74	12.1	34.9	93.3	178		85.2			7.1	7.6	0.0	0.1
1110103	0924	5520		14.8	42.3		205		79.2			10.5	7.2	2.7	0.4

DATE	TIME	NA	K	CL	BUN	CREAT	eGFR	CA	Mg	P	CRP	ESR	PCT	D-Dimer	LACTATE
1101225	1559	142	4.3		8	1.03	85.01								
1101230	1122	139	4.0	107	12	0.98	90.04	7.7							
1110103	0915	139	3.7		9	0.87		8.7							

DATE	TIME	GLUCOSE	HbA1C	CHOL	TG	HDL	LDL	KETONE	CK	CKMB	TRO-T	PT-P	APTT-P	NT-pro BNP
1101225	1559	96												
1101225	1601											10.7	28.8	
1101227	1244		4.8											

DATE	TIME	PH	BEB	SCVO2	PO2	HCO3	HGB	PCO2	TCO2
1101225	1601	7.337	2.2	30.000	19.3	29.4		56.1	31.1

- 放射線報告 :**檢驗項目 :MRI - PELVIS 報告日期 :1101230**

主　訴：
Result:Y
Finding:
MRI of pelvis pre and post iv contrast:
1. Cor. and sag. and T2WI without fat-saturtion.
2. Axial T2WI without and with fat-saturtion.
3. Axial diffusion weighted imaging (DWI) of pelvis
4. Axial T1WI without and with fat-saturtion.
5. Axial and cor. GRE T1WI post bolus iv injection of Gd-based contrast agent.
> A patient with Hx of Rectal Ca, s/p neoadjuvant CCRT, for pre-OP survey.
> Asymmetrical wall thickening at rectum with stranding of perirectal fat plane noted, compatible with rectal Ca with post-treatment change
> Mild wall thickening of urinary bladder, r/o chronic cystitis.
> No abnormal lymphadenopathy in pelvic wall.
Impression:
Compatible with Rectal Ca with post-treatment change. Suggest further management

檢驗項目 :CHEST PA VIEW 報告日期 :1110103

主　訴：
Result:N
Finding:
No cardiomegaly. No active lung lesion.
Impression:
As above description.

特殊檢查

病理報告

- 活體組織切片病理報告　手術日期：**110/12/29**　病理號：**S1044017**

診斷醫師:李欣倪 報告日期:111/01/11
Pathologic diagnosis:
1. Lymph node, pelvic, left, dissection --- Unremarkable (0/5).
2. Rectum, cut end, distal, resection --- Chronic inflammation.
3. Rectum, robot-assisted low anterior resection --- Status post-neoadjuvant CCRT with no residual invasive carcinoma. Mucosal ulceration is seen. The bilateral cut ends and perirectal lymph nodes (0/12) are free of tumor.
4. IMA root, dissection --- Unremarkable (0/1).
Ancillary study for diagnosis:
IHC stain for CDX2 done on section AB.
Prognostic and predictive factor:
1. Tumor size: No residual tumor.
2. Depth of invasion: No evidence of residual tumor.
3. Histologic grade: Not applicable.
4. Angiolymphatic invasion: Not identified.
5. Perineural invasion: Not identified.
6. Tumor deposits (discontinuous extramural extension): Not identified.
7. Macroscopic tumor perforation: Not identified.
8. Surgical Margins: a) Proximal Margin: Uninvolved by invasive carcinoma. b) Distal Margin: Uninvolved by invasive carcinoma. c) Radial or Mesenteric Margin: Uninvolved by invasive carcinoma.
9. Lymph node status: Uninvolved (0/18).
10. Extranodal involvement: Not applicable.
11. Tumor regression grading S/P CCRT: Present, no viable cancer cells (complete response, score 0).
12. Type of polyp in which invasive carcinoma arose: Not identified.
13. Additional pathologic findings: Hyperplastic polyp.
14. Pathological TNM Stage: ypT0N0(According to the eighth edition, American Joint Committee on Cancer Staging Guidelines for Tumors).
15. TNM descriptors: y (Post-treatment).

- Gross description:
  The specimen consists of 1) "left pelvic LN", a piece of tan yellow soft tissue, 4.5x3.5x1.8 cm. 2) distal cut end, a piece of tan mucosa-covered soft tissue, 2x1x0.8 cm. 3) a segment of rectum, 13.5 cm in length and 4 cm in proximal cut end circumference, 6.5 cm in distal circumference, and perirectal soft tissue. An ulcerative mucosal lesion, 1.2 x 1.2 cm, is seen, 1.5 cm from the distal cut end. An erythematous flattened area, 4x2 cm, is noted in the rectal mucosa, 3 cm to proximal cut end. Another erythematous flattened area, 1x1 cm, is noted, 4 cm to distal cut end. The remaining rectum is unremarkable grossly. 4) IMA root, a piece of tan yellow soft tissue, 2x1.5x1 cm. Representative part for section: A-B) all of specimen 1 C) all of specimen 2 D) all of specimen 4 E-F) proximal and distal cut ends in specimen 3 G-L) rectal mucosa with erythematous change M) rectum N-X) perirectal soft tissue and lymph nodes Y-AC) ulcerative mucosal lesion all for sections.
  #T-59300_2 #M-09413_2 0 1105 00SP00
  #T-C4560_2 #M-09183_2 0 1105 000000

其它

出院時情況

- 3.改門診治療

出院指示

藥名	劑量	劑量單位	用法	頻次	數量	數量單位	現況	天數	單位
Voren-K tab 25mg	1	TAB	PO	TIDPRN	15	TAB	TH	5	天
(□)Takepron OD tab 30mg	1	TAB	PO	QDAC	5	TAB	TH	5	天

出院計劃

主治醫師_2222H 林俊余 / 住院醫師_2346C 陳怡豪

正式摘要(電子簽章：000000234324839)

W102 / W102

doctorRank:B
